# Alexithymia may explain the relationship between autistic traits and eating disorder psychopathology

**DOI:** 10.1186/s13229-020-00364-z

**Published:** 2020-08-05

**Authors:** L. Vuillier, Z. Carter, A. R. Teixeira, R. L. Moseley

**Affiliations:** Department of Psychology, Bournemouth University, Poole, UK

**Keywords:** Alexithymia, Autism, Autistic traits, Anorexia nervosa, Eating disorders

## Abstract

**Background:**

Autistic people are disproportionately vulnerable to anorexia nervosa and other eating disorders (ED), and within the general population, autistic traits correlate with ED psychopathology. A putative mechanism which may underpin this heightened risk is alexithymia, a difficulty identifying and describing emotional states which is observed in both autism and ED. In two experiments with independent non-clinical samples, we explored whether alexithymia might mediate the heightened risk of eating psychopathology in individuals high in autistic traits.

**Methods:**

Our first experiment used the PROCESS macro for SPSS to examine relationships between alexithymia (measured by the Toronto Alexithymia Scale (TAS-20)), autistic traits (autism quotient (AQ)), and eating psychopathology (Eating Attitudes Test (EAT-26)) in 121 participants. Our second experiment (*n* = 300) replicated and furthered this analysis by examining moderating effects of sex and controlling for anxiety and depression as covariates. We also included an additional performance-based measure of alexithymia, the Levels of Emotional Awareness Scale (LEAS).

**Results:**

Study 1 suggested that TAS-20 scores mediated the relationship between heightened autistic traits and eating psychopathology. Replication and further scrutiny of this finding, in study 2, revealed that this mediation effect was partial and specific to the female participants in this sample. The mediation effect appeared to be carried by the difficulty identifying feelings subscale of the TAS-20, even when depression and anxiety were controlled for. LEAS scores, however, were not significantly related to autistic traits or eating psychopathology.

**Limitations:**

Cross-sectional data prevents any conclusions around the direction and causality of relationships between alexithymia, autistic traits, and eating psychopathology (alongside depression and anxiety), necessitating longitudinal research. Our non-clinical sample was predominantly Caucasian undergraduate students, so it remains to be seen if these results would extrapolate to clinical and/or autistic samples. Divergence between the TAS-20 and LEAS raises crucial questions regarding the construct validity of these measures.

**Conclusions:**

Our findings with respect to autistic traits suggest that alexithymia could partially explain the prevalence of ED in autistic people and may as such be an important consideration in the pathogenesis and treatment of ED in autistic and non-autistic people alike. Further research with clinical samples is critical to explore these ideas. Differences between men and women, furthermore, emphasize the importance of looking for sex-specific as well as generic risk factors in autistic and non-autistic men and women.

A well-documented but poorly understood relationship exists between autism spectrum conditions (ASC) and anorexia nervosa (AN), conditions both associated with long-term morbidity and increased mortality [1–4]. The health risks associated with both conditions paint a worrying picture for the disproportionate number of autistic individuals found amongst those suffering from AN [5–7], as does the fact that autism and autistic traits seem to be associated with poorer prognosis and treatment response [7–11]. It is crucial to understand the mechanisms which may explain the heightened co-occurrence of these conditions and offer important insights to treatment.

The common co-occurrence of autism and anorexia is belied by their conceptual dissimilarity. In current nosology (DSM-5 [12]), autism is recognized as a lifelong neurodevelopmental condition with genetic origins^1^ and which is sometimes accompanied by intellectual disability. The key features “persistent across multiple contexts” include difficulties in social communication and interaction, restricted and repetitive behavior, and interests. In contrast, DSM-5 conceptualizes AN as the most lethal [14] of several feeding and eating disorders (ED), an acute psychiatric illness (as opposed to a lifelong difference in functioning) characterized by restricted caloric intake, intense fear and avoidance of weight gain, and disturbed body and shape perception. Where anorexia is more commonly diagnosed in females with a typical onset in adolescence [15], autism, in contrast, is more commonly diagnosed in males [16] and is manifest in brain differences even in the first year of life.

Despite the conceptual differences between these two distinct conditions, theorists have been highlighting behavioural similarities between them for many years [17–20]. Like autistic people, individuals with anorexia exhibit detail-focused processing, impairments in aspects of executive function and cognitive flexibility, even an obsessive and perfectionistic nature [21]. Furthermore, they share in common heightened negative affectivity [21], blunted facial affect [22, 23], problems with the theory of mind and cognitive/emotional perspective-taking [21, 24, 25], and tend to have “long-standing (i.e. premorbid) patterns of interpersonal discomfort” [23], which when studied in acutely ill individuals manifest as social impairments and difficulties making friends [26]. Those with autism, too, exhibit similarities with anorexic populations, including rituals around eating and extreme food selectivity [27–29], though these may be associated with anxiety, sensory sensitivities, and gastrointestinal problems rather than the fat-phobia and weight concern of anorexia. Impaired interoception may incorporate reduced awareness of hunger and satiety [30], and autistic people may also experience distortions in body image [31].

Though ASC and AN are indeed dissociable, a substantial body of literature supports their common co-occurrence. In non-clinical populations, autistic traits and eating disorder symptomatology rise in tandem significantly correlated [32–36]. Research involving individuals with anorexia has revealed elevated autistic traits or symptomatology appear across a range of measurement tools, including screening questionnaires (such as the Autism-Spectrum Quotient (AQ) [37]) [7, 38–45], clinical interviews [46], and autism diagnostic tests [6, 47], and this consistent elevation in autistic symptomatology suggests disproportionate representation of ASC in this population [5, 6, 48, 49]^2^. Notably, this relationship seems weaker in younger children or adolescents [47, 52–54], which may in part be due to problems with parental report assessments, and parent recognition of autism in young girls [55]. Furthermore and problematically, the nature of ED is such that malnutrition can mimic the cognitive rigidity and social impairments of autism. Studies using developmental interviews or prospective approaches have, however, confirmed the presence of autistic features before the onset of disordered eating and diagnosed ED [5, 48, 56], and others have noted the persistence of social difficulties [49], problems with the theory of mind, empathy and emotion recognition [57, 58], reduced eye contact [59], detail-level processing [60–62], and cognitive rigidity [62] after recovery. Other studies, however, find these issues to recede, sometimes to the point that patients are undistinguishable from controls [63–67]. One reason for the divergence may be different definitions of ‘recovery’. However, the apparent fading away of autistic symptoms with age and/or recovery may reflect not only the influence of malnutrition but also the potentially poorer prognosis and slower or poorer treatment response of individuals with high autistic traits or diagnosed autism [7–11]—with these individuals thus more likely to be seen in adult services, this would explain the higher co-occurrence in older samples [55].

The literature thus far suggests that autistic traits or diagnosed autism are important moderators of individual risk of eating psychopathology, alongside prognosis and treatment response. However, research has thus far neglected another shared commonality seen both in autistic people and people with anorexia, one which might theoretically add extra risk and contribute to elevated AN in autism and which might strengthen or even carry the association between autistic traits and anorexic symptomatology. Alexithymia is a difficulty in identifying and verbalizing one’s emotions, a “stimulus-bound, externally orientated cognitive style” [68] characterized by a paucity of imagination and introspection. It tends to co-occur with deficits in empathy, emotion recognition, and regulation [69–72], and some suggest it reflects a more general deficit in interoception, where alexithymic people may struggle to identify and link bodily sensations and internal states with emotions [73]. Alexithymia has received increasing attention in recent years, with debate around its origin, nature, and means of assessment [74]. Regardless of conceptual differences, theorists from different approaches unanimously recognize the connections between alexithymia and pathological states or disadvantageous outcomes: it is associated with mental and physical ill-health [75, 76], psychosocial difficulties [77], self-injury [78], substance abuse [79], poorer treatment response to psychological therapy [80], including in anorexia specifically [81], and even increases the risk of suicide, especially in combination with psychiatric symptoms [82, 83].

It is estimated that approximately half of the whole autistic population (individuals with and without intellectual disability) experience comorbid alexithymia [84, 85] and that the condition is biologically based, both giving rise to symptoms traditionally labelled as ‘autistic’ and being exacerbated by the same [86]. Alexithymia is a common co-occurrence in not only anorexia but bulimia and binge-eating disorder [87]; in anorexia, it has been estimated in as many as 77% of female patients [88]. Importantly, recent efforts have been made to disentangle symptoms of autism and anorexia from those of alexithymia. Some of the central features of autism, such as the widely reported difficulties in emotion recognition, maintaining eye contact, emotion recognition, empathy, and interoception, have been linked to comorbid alexithymia but not to autism itself ([87–90] - though note 68). Likewise, emotional deficits in anorexia have been proposed to reflect comorbid alexithymia rather than the ED itself [70]. Alexithymia is related to anorexic symptomatology and social struggles in anorexia even when nutritional status is controlled for [91], though findings are mixed as to whether it persists after patients are weight-restored [87].

In a similar vein to studies disentangling the features of anorexia and autism from those of alexithymia, we query whether the relationship between the two, likewise, is in part a product of this comorbid condition. This has been precisely implied in recent, unpublished research with autistic individuals and a clinical anorexic sample, where Hobson and colleagues observed that alexithymia increased the chances of both groups meeting cut-off criteria for autism diagnosis [92]. In increasing the likelihood that anorexic individuals were more likely to meet ASC criteria on ADOS-2 items related to social affect and social insight, the authors suggest that “co-occurring alexithymia may be a potential explanation for the high proportion of patients with AN who exhibit symptoms of ASD” (p. 22). This would suggest that extant alexithymia might inflate the strength of the relationship between autism, autistic traits, and eating psychopathology. A similar idea was explored by Mansour et al. [36], who examined the mediating contribution of emotion dysregulation, negative attitudes to emotions, empathy and body dissatisfaction to relationships between the subscales of the EAT-26 (dieting, bulimia, and oral control), and autistic traits (as measured by the AQ). Whilst empathy and negative attitudes to emotions did not contribute to these relationships, the relationship between autistic traits and the dieting scale of the EAT-26 was mediated by emotion dysregulation (here measured by the Difficulties in Emotion Regulation Scale (DERS), which does in fact include two subscales linked to alexithymia: awareness and clarity of emotions) and body dissatisfaction. Though direct relationships appeared between autistic traits and the bulimia and oral control subscales, autistic traits themselves did not significantly predict scores for dieting. The authors related their findings to previous studies where comorbid emotion pathology was also seen to give rise to the apparently elevated autistic traits in an anorexic sample [93], and thus lend credence to the idea that the autism-anorexia relationship might be artificially inflated by comorbidities.

Emotion dysregulation and alexithymia are closely related concepts. For instance, some authors suggest that emotion generation and regulation are one concept all together [94], which can be observed in the way emotion regulation is sometimes measured (e.g. with the DERS). However, most authors would agree that they are unequivalent and that alexithymia probably occurs earlier in the attention to and appraisal of an emotional stimulus, whereas difficulties regulating one’s emotions would occur downstream from this at the point of an emotional response [95]. As such, to compliment the work by Mansour et al. and to investigate the proposition reflected in the findings from Hobson et al., we chose to scrutinize alexithymia as a potential mediator of the relationship between autistic traits (AQ scores) and eating psychopathology as measured by the EAT-26. In this two-pronged approach, we first tested the hypothesis that TAS-20 scores would mediate the relationship between autistic traits and eating psychopathology in a non-clinical British sample. On confirming a significant contribution of alexithymia to this relationship, a second experiment with a larger, independent sample aimed to additionally address conceptual debates around alexithymia and its measurement with the addition of a performance-based measure. In this second experiment, we also examined moderating influences of sex whilst controlling for potential confounds in anxiety and depression. A role for alexithymia in the relationship between autism and eating psychopathology, if uncovered, might have preventative value in identifying individuals at increased risk and further emphasize the need to consider this variable in therapeutic interventions and predicting outcomes [81, 87].

## Experiment 1: methods

### Participants

A total of 121 participants (*n* = 101 females, mean age = 24.3, SD = 8.4; age range = 18–64 years old) were recruited from the student population of Bournemouth University and from social media. Informed consent was obtained according to procedures approved by the Bournemouth University ethics committee. Participants were rewarded with 0.5 course credit (if they were students) or the chance to win a £50 Amazon voucher (external participants) for their time in the study.

Participants were not excluded on the basis of psychiatric diagnoses. Fifty-two (43%) participants reported an additional diagnosis, with the most common being depression (11 participants), anxiety (15 participants), or combined depression and anxiety (21 participants); additional diagnoses included OCD (3 participants), PTSD (2 participants), personality disorder (4 participants), psychosis (1 participant), and ADHD (1 participant). Two participants reported a diagnosis of an autism spectrum condition, and six (no overlap) reported an eating disorder. As these conditions are likely to appear within the normal distribution of the population, these participants were included in the analysis, but a secondary analysis confirmed that results remained significant without them.

### Materials and procedure

The study was hosted on an online platform (Qualtrics). After providing some brief demographic details including details of any neurodevelopmental conditions, and/or current, previous, or suspected ED, participants were free to work through the three questionnaires below in their own time. In order of completion, these were as follows.

#### The Autism-Spectrum Quotient (AQ) [37]

This measure rests on the theoretical proposition that autism spectrum conditions occur at the extreme end of a “continuum of socio-communicative disability” (p.6) encompassing the normal distribution. It has been widely translated and extensively used in the general population and those with diagnosed autism [96–98], and boasts strong psychometric properties, including internal consistency and test-retest reliability. A cut-off score of 26 is recommended when the measure is used for screening purposes in non-clinical groups.

#### The Toronto Alexithymia Scale (TAS-20) [99]

A short self-report measure of 20 statements, the TAS-20 can be subdivided into three factors, reflecting identification of one’s own emotional states (difficulty identifying feelings: DIF) (e.g. “I am often confused about what emotion I am feeling”), the ability to verbally describe emotional states to others (difficulty describing feelings: DDF) (e.g. “It is difficult for me to find the right words for my feelings”), and an inclination away from introspection and towards externally orientated thinking (EOT) (e.g. “I prefer talking to people about their daily activities rather than their feelings”). Scores above 61 indicate a clinically substantive level of impairment. The TAS-20 boasts good internal consistency and test-retest reliability [100, 101], being the most popular and most highly translated self-report tool for alexithymia, and has been the most extensively used in ED research [87].

#### The Eating Attitudes Test (EAT-26) [102]

This measure was chosen as one of the most widely used measures of eating psychopathology [103] and comprises subscales measuring restrictive eating (dieting), bulimic behaviours, and food preoccupation (bulimia and food preoccupation), and behaviours around controlling amount and ways of eating (oral control). The specificity of these subscales to different ED is however weak [103, 104], so only the total score of the 26 items was analysed (excluding responses to the items on behaviours, current height and weight) and must be conceptualized as a measure of eating psychopathology. The test boasts strong psychometric properties, including construct validity, test-retest reliability, and discriminant ability between individuals with current ED, subthreshold symptoms, and recovered ED [105, 106]. Scores above 20 indicate the presence of eating psychopathology.

The first experiment employed only total scores for each measure, all of which achieved satisfactory alpha levels (see Supplementary materials). After participants had completed the three scales in the order listed above, they were debriefed and thanked for their time.

### Analysis

Whilst multiple approaches exist for mediation analysis, we adopted the bootstrapping (resampling with replacement) method employed by the PROCESS macro for SPSS (version 3) [107]. This powerful method can detect mediation or moderation effects even in small samples, with a smaller likelihood of type I errors and freedom from assumptions of normality in the sample distribution [107]. With the number of bootstrap samples set to 5000, we analysed relationships between variables in accordance with PROCESS Model 4, where autistic traits (X) would exert an influence on eating disorder symptomatology (Y) via the intervening, mediator variable of alexithymia (M). On computing the correlation coefficients between X and M (path *a*), M and Y (path *b*), and X and Y (path *c*, the total effect [which includes the influence of M]), the direct effect of X on Y (path *c’* [c prime]) controls for the presence of the mediator. The indirect effect (*ab:* the path coefficients of *a* and *b*) thus quantifies the strength or influence of the mediator on the X–Y relationship.

We report the data in accordance with both the usual alpha level of *p* < .05 and a more conservative alpha level of *p* = .017 after controlling for multiple comparisons.

## Experiment 1: results and interim discussion

Descriptive statistics (see Table 1) showed that despite some variation in autistic traits, eating psychopathology, and alexithymia, the average scores of the sample were below clinical or diagnostic cut-offs for each of these variables. There were however some exceptions where participants indicated notably higher levels of autistic traits, eating psychopathology, or clinical alexithymia.

**Table 1:** Experiment 1: average scores, followed by standard deviation (brackets) and range (italics) for all participants on the Autism-Spectrum Quotient (AQ), Eating Attitudes Test-26 (EAT-26), and Toronto Alexithymia Scale-20 (TAS-20). The second column represents the number of participants who scored at or above cut-offs for autistic traits (26 for the AQ), eating psychopathology (20 for the EAT-26), and alexithymia (61 for the TAS-20)

	All participants (*n* = 121)	Participants scoring at/above cut-offs (*n*)
Autistic traits (AQ)	16.0 (8.0), *45*	11
EAT-26 total	12.2 (11.3), *56*	25
TAS-20 total	47.8 (12.5), *56*	19

**Fig. 1:**
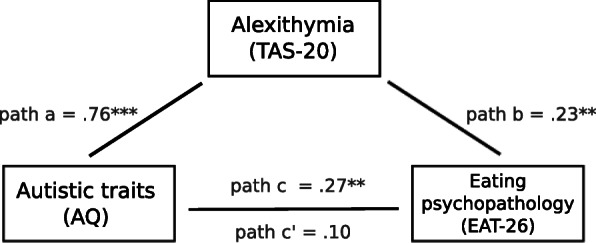
Experiment 1: regression coefficients for the relationship between autistic traits (AQ) and alexithymia (TAS-20) (path a), between alexithymia and eating psychopathology (EAT-26) (path b), between autistic traits and eating psychopathology including the contribution of alexithymia (path c—the total effect), and between autistic traits and eating psychopathology controlling for the mediating effect of alexithymia (path c’). *P* values are reflected by asterisks where *p* < .001 is depicted with three asterisks, and *p* < .01 is depicted with two asterisks

As shown in Fig. 1, significant relationships existed between autistic traits and alexithymia (path a: *b* = .76, *p* < .001; *R*^2^ = .24, *F* (1, 119) = 37.4, *p* < .001), and between alexithymia and eating psychopathology (path b: *b* = .23, *p* = .013). The total effect (path c), that is the relationship between autistic traits and eating psychopathology without controlling for the effect of alexithymia was significant (*b* = .27, *p* = .036; *R*^2^ = .04, *F* (1, 119) = 4.51, *p* = .036; though this relationship did not meet our more stringent corrected alpha level). However, when controlling for the presence of alexithymia, the direct relationship between autistic traits and eating psychopathology (path c’) became non-significant (*b* = .10, *p* = .507); the indirect effect of alexithymia on the relationship between autistic traits on eating psychopathology (*ab*) was .18, a significant effect (CI .032, .347). This shows that alexithymia accounted for 18% of the effect of autistic traits on eating psychopathology.

The complete disappearance of the significant relationship between autistic traits and eating psychopathology, upon controlling for TAS-20 scores, suggests a full mediation effect: that the aforementioned relationship was dependent on the presence of alexithymia. However, a degree of confidence in this finding necessitated replication for several reasons. Firstly, it is important to recognise that due to the differing biological and sociocultural influences on males and females, there are likely both generic and sex-specific factors at play in the pathogenesis of eating disorders [108]. Where studies of autism and of anorexia tend to study predominantly male or female samples respectively, one goal of our replicative second study was to examine potential moderating influences of sex, in order to identify if alexithymia is an important contributor to the autism-anorexia relationship for both males and females.

A second goal of the second experiment was to deconstruct and further elucidate the link between autistic traits and eating psychopathology, and the mediating role of alexithymia in this relationship. We did this in two ways. Initially, we examined whether any one particular element of the alexithymia construct as conceptualized by Bagby and colleagues was especially important to the relationship between autistic traits and eating psychopathology. The individual subscales of the TAS-20 (DIF, DDF, and EOT) were henceforth scrutinized as independent mediators between AQ and EAT-26 scores.

As an additional step in our deconstruction of the alexithymia construct, we considered how other measures of alexithymia might operate within the AQ–EAT-26 relationship. The TAS-20 is perhaps the most popularly used of self-report measures for alexithymia, but an inherent fallacy has been argued to exist in self-report measures which require highly alexithymic participants to accurately report how they feel [109, 110]. Moreover, the TAS-20 may more reflect people’s beliefs about themselves rather than provide an accurate representation of their ability [111]. It is known to detect neuroticism, shame, and general negative affect, as well as somatic or medically unexplained symptoms [112–115]. This issue is averted via behavioural measures which can corroborate poorer ability to identify, differentiate between, and describe emotions. Importantly, two studies investigating patients with anorexia [116, 117] found a disparity where high self-reported levels of alexithymia (TAS-20) were belied by scores comparative to controls in one such measure, the Levels of Emotional Awareness Scale (LEAS: [118]). They note that the self-report measures such as the TAS-20 require judgements of self-efficacy in the identification and describing of emotions, and in hence involving self-evaluation of one’s own emotion-processing abilities, may be distorted by genuine lack of awareness (alexithymia) or by low self-image^3^. In accordance with this view, the LEAS has, indeed, been seen to be only weakly correlated, if at all, to the TAS-20 [111]. In an attempt to elucidate what precisely drives the mediating effect of alexithymia on the relationship in question, and further to check any disparity between self-reported alexithymia and behavioural emotion identification and descriptive skills, we thus also explored LEAS scores as a mediator in the relationship between autistic traits and eating psychopathology.

A third and related goal pertained to the need to control for the potentially confounding variables related to alexithymia. As anxiety and depression contribute substantial variance to TAS-20, and likewise to LEAS scores to a lesser extent [119, 121, 122], our second experiment followed recommendations [123] to include these as covariates in order to disentangle their contribution from that of alexithymia.

## Experiment 2: methods

### Participants

An independent sample of 300 participants (*n* = 237 females, mean age of total group = 20.7, SD = 5.6; age range = 18–60 years old) was recruited opportunistically from the student population of Bournemouth University and from social media. Ethical procedures were followed and participant compensation awarded as in experiment 1.

As before, participants with psychiatric diagnoses were not screened out. Ninety-five participants (31.7%) disclosed that they had a current or historic psychiatric diagnosis, most commonly depression (21 participants), anxiety (30 participants), or combined depression and anxiety (29 participants); other diagnoses were OCD (6 participants), PTSD, ADHD or personality disorder (2 participants for each), and psychosis (1). Seventeen participants had formally been diagnosed with an eating disorder (anorexia nervosa [9], bulimia nervosa [5], binge-eating disorder [2], or eating disorder not otherwise specified [1]). Four participants had an autism spectrum diagnosis (three of the four also had an eating disorder: bulimia, anorexia, and binge-eating disorder respectively)—again, analyses were re-run without these participants to confirm that results remained consistent.

### Materials and procedure

As in experiment 1, participants first completed the TAS-20, the AQ, and the EAT-26. They were then asked to complete the following, additional measures:

#### The Levels of Emotional Awareness Scale, short form (B) (LEAS: [118])

This measure assesses awareness of emotional states and the depth and complexity of an individual’s emotion vocabulary. It was originally based on a five-tiered developmental model of emotional awareness, where higher scores reflect access to and ability to verbalize more sophisticated blends of emotion that might differentiate between the self and one other. In accordance with the relationship of alexithymia to mental and physical ill-health, lower scores in the LEAS, too, have been associated with a number of pathological states including somatic disorders, depression, PTSD, substance abuse, and even ED [119, 124–127]. With the ten vignette version, participant scores ranged between 0 and 50, with the normative average being 31 (see Supplementary materials for more, including details of scoring).

#### The Patient Health Questionnaire (PHQ-9 [128])

This short screening measure of state depression (within the last 2-week period) has received extensive clinical usage and possesses strong psychometric properties [129]. Scores of 5–9, 10–14, 15–19, and 20–27 indicate mild, moderate, moderately severe, and severe depression respectively. A normative score of 3.2 is seen for people in the 18–29 age bracket [130].

#### The Beck Anxiety Inventory (BAI [131])

This short screening test for state anxiety (within the last week) has likewise received popular clinical use and extensive validation of its properties [132]. Scores of 0–7 indicate minimal anxiety; scores of 8–15, 16–25, and 30–63 indicate mild, moderate, and severe anxiety respectively. The normative average for participants in the 18–44 age bracket is 7.3 [133].

After completing the 6 questionnaires, participants were thanked and debriefed. Reliability scores were again checked for this sample (see Supplementary materials) and proved satisfactory in all measures with the exception of the EOT (externally orientated thinking) subscale of the TAS-20, which has indeed been shown to be the least reliable subscale in previous research [101].

### Analysis

Analysis for our second experiment utilized PROCESS Model 59 for moderated mediation. Herein, we first replicated the mediation analysis of total AQ scores (X), total TAS-20 scores (M), and total EAT-26 scores (Y), whilst examining moderating effects of sex (W) on each pathway. As a second step and towards the goal of deconstructing the alexithymia construct, we replaced the single TAS-20 total first with the subscales of the TAS-20 (DIF, DDF, and EOT) as parallel mediators, and then with total LEAS score, all the while retaining sex as a moderating factor.

Unfortunately, a technical error meant that we obtained PHQ and BAI scores for only 223 of the 300 participants (40 males). As such, we repeated our deconstructed mediation analysis above (first for the TAS-20 subscales, then for the LEAS) for this subset of participants whilst controlling for depression and anxiety.

As before, for each mediation analysis, results were interpreted in accordance with standard alpha levels (*p* < .05) and checked against a more stringent corrected alpha level of *p* = .017 for analyses with the TAS and LEAS total, and *p* = .007 for the mediations using the three subscales of the TAS-20.

## Experiment 2: results

Descriptive statistics for our female and male participants (see Table 2) revealed that on average (with a few exceptions), our typically developing participants were below suggested cut-offs signifying diagnostic levels of autistic traits, eating psychopathology, and alexithymia.

**Table 2:** Experiment 2: average scores, followed by standard deviation (brackets) and range (italics) for male and female participants on the Autism-Spectrum Quotient (AQ), Eating Attitudes Test-26 (EAT-26), Toronto Alexithymia Scale-20 (TAS-20 total and subscales), and Levels of Emotional Awareness Scale (LEAS). The fourth column depicts the number of participants (female/male) who scored above cut-offs on the AQ (cut-off: 26), the EAT-26 (cut-off: 20), or the TAS-20 (cut-off: 61), and below the general population average for the LEAS (31)

	All participants (*n* = 300)	Female (*n* = 237)	Male (*n* = 63)	Participants scoring at/above cut-offs, or below LEAS average: (*f*) *n*/(*m*) *n*)
Autistic traits (AQ)	16.7 (7.2), *46*	16.4 (6.8), *42*	17.8 (8.6), *43*	28/7
EAT-26 total	11.7 (12.4), *75*	12.7 (13.2), *75*	7.8 (7.8), *43*	53/15
TAS-20 total	50.2 (12.7), *65*	50.1 (12.9), *65*	50.5 (12.2), *47*	52/4
TAS-20 DDF	14.2 (5), *20*	14.2 (5), *20*	14.5 (5.1), *19*	–
TAS-20 DIF	16.7 (6.7), *26*	16.9 (6.9), *26*	16.1 (6.1), *22*	–
TAS-20 EOT	19.2 (4.1), *24*	19 (4.1), *24*	19.8 (4.2), *18*	–
LEAS	31.8 (3.7), *23*	32.0 (3.7), *23*	30.8 (3.7), *15*	94/33

**Fig. 2:**
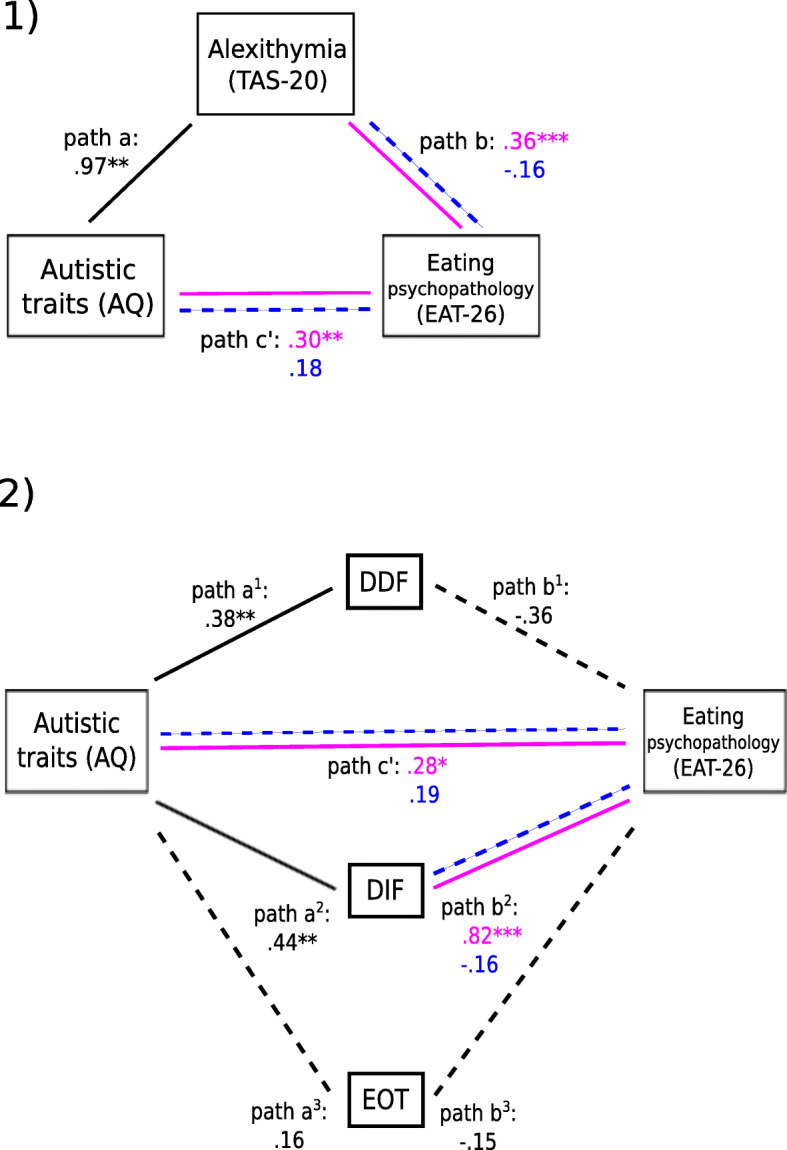
Experiment 2: Part 1 depicts relationships and mediation effects between autistic traits (AQ total), alexithymia (TAS-20 total), and eating psychopathology (EAT-26 total), with sex as a moderator. Relationships unaffected by sex are depicted in black; where sex did exert a moderating effect, blue and pink are used to depict the nature of relationships for male and female participants respectively. Part 2 depicts relationships between autistic traits (AQ total) and eating psychopathology (EAT-26 total) with the subscales of the TAS-20 (DDF, DIF, EOT) as parallel mediators and sex as a moderator, using the same colour scheme to depict where and how relationships were moderated by sex. In both parts, *p* values are reflected by asterisks where *p* < .001 is depicted with three asterisks, *p* < .01 is depicted with two asterisks, and *p* < .05 is depicted with an asterisk

In a replication of the analysis from experiment 1, with total scores from the AQ, TAS-20, and EAT-26, significant relationships existed between autistic traits and alexithymia (path a: *b* = .97, *p* < .0044; *R*^2^ = .26, *F* (3, 296) = 34.25, *p* < .001), and between alexithymia and eating psychopathology (path b: *b* = − .68, *p* = .0342). Sex exerted no significant effect or moderating influence on path a, but exerted a significant effect on the model predicting eating psychopathology (*b =* − 22.79, *p* = .0011, reflecting higher scores in females) and a significant moderation effect (*b =* .52, *p* = .0024) on the relationship between eating psychopathology and TAS-20 scores. This pathway (b) was significant for female (*b* = .36, *p* < .001) but not male participants (*b* = − .16, *p* = .3028). A significant index of moderated mediation (*b* = .46, CI .21, .77) indicated that sex moderated the critical relationship between autistic traits and eating psychopathology, and breakdown of this effect revealed a partial mediation for female participants, with both a significant direct effect of autistic traits on eating psychopathology (path c’: *b =* .30, *p* = .0128), and a significant indirect effect via TAS-20 scores (*ab* .31, CI .16, .48). For male participants, these direct (*b* = .18, *p* = .41) and indirect effects (*ab* − .14, CI − .41, .04) were non-significant (Fig. 2, part 1).

Next, the subscales of the TAS-20 were entered as parallel mediators (Fig. 2, part 2). This secondary analysis revealed significant relationships between autistic traits and DDF (path a^1^: *b* = .38, *p* = .0074) and between autistic traits and DIF (path a^2^: *b* = .44, *p* = .0183), but not between autistic traits and EOT (path a^3^: *b* = .16, *p* = .1926). DIF and DDF were seen to furthermore be strongly correlated with each other (*b* = .72, *p* < .001), as to a weaker extent were DDF and EOT (*b* = .35, *p* < .001), and DIF and EOT (*b* = .21, *p* = .001)—but sex exerted no significant main or moderation effects. A main effect of sex emerged in the model predicting eating psychopathology (*b* = − 16.81, *p* = .0412; *R*^2^ = .24, *F* (9, 290) = 9.95, *p* < .001), but a significant moderation effect (*b* = .97, *p* = .0105) was seen only on the relationship between DIF and eating psychopathology (path b^2^), which was significant for females (*b* = .82, *p* < .001) but not for males (*b* = − .16, *p* = .6481). Pathways between DDF (path b^1^: *b* = − .36, *p* = .6688) and EOT (path b^3^: *b* = − .15, *p* = .8394) and eating psychopathology were non-significant for all participants and unaffected by sex. The direct effect (c’) of autistic traits on eating psychopathology, as above, remained significant for females (*b* = .28, *p* = .0216) but not for males (*b* = .19, *p* = .3918). For females, this relationship was partially mediated by DIF, as reflected in a significant indirect effect (*ab*: *b* = .36, CI .21, .54); with a significant index of moderated mediation (*b* = .43, CI .16, 74), this indirect effect was non-significant for males (*ab*: *b* = − .07, CI − .32, .13). The indirect effects of DDF and EOT were non-significant for males and females.

We next examined total LEAS score as a mediator. Whilst the overall model predicting LEAS scores was marginally significant (*R*^2^ = .03, *F* (2, 296) = 2.66, *p* = .0483), the relationship between autistic traits and LEAS scores was not (path a: *b* = .12, *p* = .296); there was instead a main effect of sex where female participants tended to outperform males (*b* = 2.66, *p* = .0328), though no significant moderation. In the significant overall model predicting eating psychopathology (*R*^2^ = .12, *F* [5, 294] = 7.88, *p* < .001), there was no significant relationship between LEAS scores and eating psychopathology (path b: *b* = .62, *p* = .46), and no significant direct effect of autistic traits on eating psychopathology (path c’: *b* = .53, *p* = .15). A significant moderation effect (*b* = .57, *p* = .0066) revealed that this direct effect was in fact significant for women (*b* = .61, *p* < .001), as seen above. With a non-significant index of moderated mediation (*b* = .01, CI − .04, .10), the indirect effect of LEAS scores on the relationship between autistic traits and eating psychopathology (*ab*) was non-significant for men (*b* = − .01, CI − .08, .04) and women (*b* = .00, CI − .02, .05). On checking for relationships between the LEAS and the TAS-20 (and its subscales), all relationships were well below significance.

We then analyzed a subset of the same participants in whom we could control for depression and anxiety (see Supplementary materials for descriptive statistics and full statistical notations). With BAI and PHQ scores entered as covariates alongside DDF, DIF, and EOT, the direct effect of autistic traits on EAT-26 scores was non-significant (*b* = − .03, *p* = .82; *R*^2^ = .33, *F* (6, 216) = 17.485, *p* < .001), with depression (*b* = .71, *p* < .001), anxiety (*b* = .15, *p* = .0468), and DIF (*b* = .46, *p* = .0079) significantly contributing to the model. As before, only DIF exerted a significant indirect effect (*b* = .11, CI .0280, .2064) mediating the relationship between autistic traits and eating psychopathology. As in our previous analysis with LEAS total as a mediator, there was no significant direct effect of autistic traits on eating psychopathology and no mediating effect of LEAS scores, but significant contributions from anxiety and depression to the model predicting eating psychopathology.

## Discussion

Understanding the commonalities which link autism and anorexia nervosa seems imperative, given that autistic individuals and those with high autistic traits seem disproportionately over-represented in populations with this deadly disorder [5, 7, 17–20, 38–41, 43, 45, 48, 49, 55, 91] and seem to tend towards a poorer prognosis and treatment response [7–11, 55]. We scrutinized the relationship between autistic traits and eating psychopathology with a focus on one putative mediating factor that might hypothetically serve as a risk factor for ED in autistic people and those with high autistic traits: levels of alexithymia. We corroborated the relationship between autistic traits and eating psychopathology which has been previously observed with these same measures [32–36], but three new findings are of especial interest. These include (a) a complete or partially mediating role of alexithymia as reflected in TAS-20 scores, notably the difficulty identifying emotions aspect, in the relationship between autistic traits and eating psychopathology; (b) the influence of several other variables on this relationship, including sex, anxiety, and depression; and (c) the divergence between self-report (TAS-20) and performance-based (LEAS) measures of alexithymia. We will discuss these three major findings, their implications, and complexity of interpretation, in turn.

### Alexithymia: a bridge from autistic traits to eating psychopathology?

That controlling for alexithymia (TAS-20 scores) as a mediator resulted in complete (with a mixed sample, experiment 1) or substantive (females only, experiment 2) loss of significance in the relationship between autistic traits and eating psychopathology is a startling finding in several ways. Initially, it calls into question the well-established relationship demonstrated in previous studies with these very measures. The full mediation effect seen in experiment 1 suggested that autistic traits exert their influence on eating psychopathology not directly, but entirely via alexithymia. Attempted replication of this effect, in experiment 2, added extra definition to this finding, suggesting that the mediation effect was in fact partial, contributed to by other factors, and present in females alone. Heightened autistic traits were still associated with heightened eating psychopathology when the effect of TAS-20 scores was controlled for, but alexithymia accounted for almost a third (31%) of the variance in this relationship, such that autistic traits also exerted their influence on eating psychopathology via alexithymia. The effect size of a mediator must be interpreted with caution, as it can be distorted by direct and indirect effects that cancel each other out [134] and by the potential presence of other, unmeasured variables that affect all three variables and the relationships between them. We controlled for two of these in depression and anxiety, and in doing so confirmed that one particular facet of alexithymia, DIF, retained its mediation effect on the relationship between autistic traits and eating psychopathology.

Casting doubt on previous evidence of a direct relationship between autistic traits and eating psychopathology, our data is redolent of the recent findings from Hobson et al. [92]. These authors found that anorexic patients were more likely to reach diagnostic cut-offs for autism if they also exhibited comorbid alexithymia (measured in TAS-20 scores). In so far as alexithymia is concerned, one possibility is that comorbid alexithymia may make autistic features more obvious or clinically severe (as in Hobson and colleague’s study, alexithymia seemed to inflate autism diagnostic scores in an autistic sample, too), thus artificially boosting the relationship between autistic traits or diagnoseable autism and eating psychopathology. This would be even more important to control for if, as mentioned before, both alexithymia and autistic traits are a product of the ED disease state. This interpretation also seems fitting with data from Westwood, Mandy and colleagues [6], where in anorexic patients, a relationship was seen between autistic symptoms (as measured by the ADOS-2 [135]) and TAS-20 scores, but not with eating psychopathology directly: the authors suggest “it is possible that symptoms such as these may mediate the relationship between AN and ASD, causing individuals to appear autistic as a secondary effect of other symptoms” (p.7).

Another potential interpretation of the mediation effect, one more clinically relevant, is that autistic individuals, or individuals with high autistic traits, are only at greater risk of eating psychopathology if they also exhibit higher levels of alexithymia; that having greater autistic traits alone does not confer this heightened risk. This interpretation is contraindicated, however, by the partial mediation effect seen in experiment 2, which suggests a model wherein autistic individuals and those with high autistic traits may still be at heightened risk for eating psychopathology by virtue of their autistic traits alone (which also dispose them to other potential contributors, like depression and anxiety), but these autistic traits might exert an additional pathway to eating psychopathology through their close relationship with alexithymia. Several mechanisms have been posited as to how autistic traits or diagnoseable autism could heighten the risk of eating psychopathology: a strong “systemizing” drive that would lend itself to meticulous monitoring of caloric intake [45]; increased likelihood of digestive problems and food sensitivities, and cognitive rigidity and narrow focus that could further exacerbate selective eating or food refusal [21]; increased likelihood of poorer body image [31], higher weights [136], and thus potential weight-based discrimination alongside the generally heightened risk of bullying [137]; and a failure to pick up the typical social rules and reinforcement around eating [21]. The ways that alexithymia could confer heightened risk have been extensively studied in the ED domain. “Secondary” alexithymia, that which arises from environmental influences rather than being purely dispositional [68], might result from an emotionally repressive upbringing and lead to distress associated with experiencing an emotion, avoidance, and maladaptive coping strategies [138]. Whether dispositional or environmental, alexithymia has been suggested to increase risk of psychopathology as part of greater difficulties with emotion regulation, including having limited strategies; poorer emotional awareness and differentiation (the alexithymia aspect); poorer inhibition of impulses, especially when distressed; and lower likelihood of being accepting and tolerant of own emotions [139, 140]. Alexithymia is also suggested to exacerbate the social difficulties [91] which some theorists place central to anorexia and other ED [141]. Alexithymia’s contribution to autistic symptomatology itself is debated, but it is posited to exacerbate (if not cause) deficits in emotion regulation, emotion recognition, and empathy, and thus compound social difficulties [142–145]. As such, it seems perfectly logical that co-existing alexithymia alongside autism or autistic traits would increase (as opposed to cause) an individual’s risk of developing eating psychopathology. In qualitative research, alexithymia has indeed been identified by autistic people themselves as one risk factor for their ED, alongside other elements of being autistic [146]. These qualitative comments and our own quantitative findings, if validated by future research, would corroborate the marked clinical import of identifying comorbid alexithymia in autistic people [85].

### Moderators and covariates in the AQ–(TAS-20)–EAT-26 relationship

#### Sex

Our second experiment allowed us to examine the impact of several other variables and their influence on alexithymia, our focal mediator. In large part because of the female predominance in ED [15], studies investigating autism and autistic traits in anorexic or other ED patients have involved female samples with few or no male participants [5–7, 38–42, 45–49, 52, 53]. Even studies exploring the autism-anorexia link in the typically developing, healthy population have tended to adjust or control for effects of sex in correlation analyses [32, 33, 36], rather than actively examining them. The involvement of autistic traits (and indeed alexithymia) in male disordered eating reflects an important gap in our knowledge and motivated our interest in sex as a moderator.

In so far as TAS-20 scores are concerned, a major finding from our analysis was the sex specificity of certain relationships. Inclusion of sex as a moderator revealed that relationships between eating psychopathology and TAS-20 (total and subscale scores; path b) were moderated by sex. The mediation of the central relationship under investigation (between autistic traits and eating psychopathology) was also moderated by sex, as reflected by a significant index of moderated mediation—alexithymia partially mediated this relationship for female participants, but not for males. The connection between autistic traits and alexithymia corroborates the general prevalence of alexithymia in both male and female autistic people [84, 86], but the lack of relationship between eating psychopathology and alexithymia was surprising, given the links demonstrated between these phenomena in women [87], and intriguing, as to our knowledge, there are no studies demonstrating alexithymia as a prominent feature of male ED.

Our male sample was small, and although the average mean, standard deviation, and range of scores in the TAS-20 and the AQ were comparable to the female group, the mean EAT-26 score was markedly lower. Participants were more closely clustered around the average score, with a smaller range and standard deviation, and so it is possible that the limitations of this sample were responsible for the moderating effect of sex. The bootstrapping method is generally robust to violations of normality and suited to small sample sizes [107, 147], so the possibility that relationships between autistic traits, eating psychopathology, and alexithymia do differ in males is still plausible until replicated in a larger sample. This interpretation is bolstered by recent, yet unpublished data from the Tchanturia group, who examined eating psychopathology in autistic men [148]. Though significant relationships between the subscales of the Eating Disorder Examination Questionnaire (EDE-Q) and the AQ-short were seen, regression revealed that anxiety and BMI fully explained the variance in eating, shape, or weight concern, where autistic traits did not. Given the absence of a relationship between autistic traits and restrictive symptoms and that higher BMI in the male autistic participants was instead associated with greater eating psychopathology of the binge-eating type, the authors suggest that “restrictive eating disorders might be less relevant to autistic men compared to women” (p. 11).

Like our own findings, this suggestion certainly requires replication and validation, particularly as the study involved a community rather than clinical sample. There is a vital need, generally, to attend to the central and peripheral factors that differentiate male from female ED. However, given that prevalence of ED seem more balanced in neurodiverse men and women than in the general population [149], it is also crucial to investigate sex-specific mechanisms driving the increased risk for ED in autistic people and those with heightened traits. Considering the downplaying of the relationship of both alexithymia and autistic traits to eating psycholopathology in our male sample, it seems entirely plausible that different risk factors in men and women may make the co-occurrence of autism and eating disorders more likely. This lack of relationship between ED and alexithymia might explain why there was no relationship between ASD and ED in our male sample, strengthening the case for alexithymia being a mediator of this relationship. However, due to limitations of our sample described above, this is only hypothetical at this stage and further research is needed with a larger male sample to confirm these results.

#### Anxiety and depression

In their unpublished study of eating psychopathology in autistic men, Kinnaird et al. [148] highlight anxiety as a putative mechanism of importance; as this most consistently predicted scores on the EDE-Q, the authors speculate that anxiety might be a risk factor that increases autistic vulnerability to eating disorders. The importance of anxiety as “one developmental pathway” to AN [150] has been recognized in non-autistic people—but just as with alexithymia, malnutrition in individuals with disordered eating makes it difficult to decipher any pathogenic role of anxiety or depression. Like alexithymia and malnutrition, anxiety and depression artificially inflate autistic symptomatology [39, 48, 55]. Furthermore, alexithymia is intricately linked to depression and anxiety in ED and non-ED populations [151–154]. Though the present study could not speak to causal primacy between any of these constructs, we nevertheless attempted to disentangle any individual variance contributed by alexithymia as a mediator from potential contributions of depression and anxiety, as recommended in recent review [123]. The data suggested independent mediating effects of depression, anxiety, and the DIF subscale of the TAS-20 underpinning the relationship between autistic traits and eating psychopathology—that these, indeed, wholly explained the relationship, further casting doubt on the direct relationship between autistic traits and eating symptomatology, and highlighting the importance of controlling for these variables in future studies. That DIF stood out from the TAS-20 subscales is concordant with data from Torres et al. [154] in a clinical sample. Whilst depression wholly mediated the relationship between DDF and a diagnosis of AN, these authors found a relationship between DIF and AN independent of depression. It is necessary at this point to consider alexithymia and its assessment in more depth, whereby we shall revisit the point around depression and anxiety.

### Decoding alexithymia: self-report vs. performance measures

Our discussion thus far pertains to self-reported alexithymia as measured by the TAS-20, but there is theoretical confusion and debate around the nature of alexithymia, with different groups producing measures based on their theoretical positions [74]. This emerged in our analysis with the discrepancy between self-report and behavioural measures of alexithymia: the former related to both autistic traits and eating psychopathology and a significant mediator between the two, the latter unrelated to autistic traits, eating psychopathology, or even anxiety and depression. Further theoretical deconstruction of these measures and the construct is thus necessary in order to understand this difference and identify where heightened risk may lie.

The “gold standard” of alexithymia measurement, the TAS-20, rests on the perspective that alexithymia is a dimensional construct with subclinical levels [155], encapsulating four factors: difficulty identifying feelings (DIF), difficulty describing feelings (DDF), externally orientated thinking style (EOT), and constricted imagination (not represented as an independent factor in the TAS-20). In this approach, EOT and constricted imagination are associated with operative thinking, i.e. thinking that is functional and stimulus-bound such that emotional states are not attended to, and DIF and DDF with poor appraisal of emotions [74]. In experiment 2, the mediation effect of total TAS-20 score was clearly carried by the DIF factor even when anxiety and depression were controlled for. This is redolent of the aforementioned suggestions that poor emotion awareness increases psychopathology in conjunction with poor emotion regulation skills [139, 140]. Where DIF might reflect a problem with initially recognizing and appraising an emotion, emotion dysregulation—suggested by Mansour and colleagues to mediate the relationship between AQ and the dieting subscale of the EAT-26 [36]—would occur downstream or potentially even consequentially from this [101]. The lower reliability of EOT and its lack of relationship with eating disorders are consistent with previous research [101, 123, 138], explaining further why EOT did not emerge as a mediator in this analysis.

The TAS-20 is far from universally supported as a valid measure of alexithymia, hence our inclusion of a performance-based measure of difficulty identifying, differentiating, and describing emotions. One aforementioned criticism frequently levelled at the TAS-20 is its confounding relationship with negative affect and comorbid psychopathology like depression and anxiety (which may affect the LEAS less [117–119]—though see 223). We too found that depression and anxiety were related to the DDF and DIF subscales of the TAS-20 (and not to the LEAS). While some have found DIF especially related to distress [115], others studying the construct with relation to AN have identified contributions of DIF which are distinct from those of depression, at least [154]. Our data supports this view, as DIF remained a significant mediator once depression and anxiety were controlled for. While controlling for anxiety and depression may be warranted, the authors of the TAS-20 recently suggested that though scores on the TAS-20 may fluctuate in a minor way due to transient states of depression and anxiety, higher or lower levels of trait alexithymia can still be detected with relative stability across an individual’s lifespan. Furthermore, these authors conceptualize the relationship between alexithymia and negative affect as a fundamental aspect of their theoretical model, where alexithymia would be expected to give rise to negative affect. As such, the authors suggest that where shared variance is seen between alexithymia, depression, and anxiety, this is less of a concern than a natural reflection of the causal primacy of alexithymia in generating psychopathology, suggesting controlling for such factors may not be necessary after all.

Another motivation for our inclusion of the LEAS is that it bypasses the fundamental confound that profoundly alexithymic individuals should, by nature of the construct, have difficulty with self-report [109, 110, 118, 156]. In perfectionistic groups tending towards low self-worth—such as individuals with eating disorders—it may also be problematic to require self-evaluation of emotion-processing skills. This is the precise explanation offered by studies which found people with eating disorders to perform comparably to controls on the LEAS even whilst reporting higher alexithymia on the TAS-20 [114, 115]—though see [117, 153]. To our surprise, there were no correlations between our variables of interest and performance on the LEAS. The comparative scarcity of LEAS research in eating disorders is in contrast to the complete lack (to our knowledge) of LEAS research in autism, but the difference between results in our analysis is problematic to the assumption that the LEAS and TAS-20 capture to the same theoretical construct. Consistent with the lack of association between them in our data, one recent meta-analysis suggests relationships between scores in the two tests are negligible at best [111], suggesting it may not afterall capture related concepts.

Whereas the full alexithymia construct is commonly understood to encapsulate the aforementioned limited imagination and externally orientated thinking style, the LEAS has a narrower focus in “emotional awareness” which should encapsulate DIF and DDF but not EOT or restricted imagination. Unlike the TAS-20, the LEAS directly tests emotion differentiation, the ability to “verbally characterize their emotional experiences with granularity and detail” ([94], p. 10), and so is heavily dependent on linguistic ability. The LEAS also loads on cognitive and affective theory of mind [157], which Bird and colleagues have argued are distinct from the TAS-20 [142, 144, 158]. As such, Lane et al. suggest that where the LEAS taps “a person’s ability to make mental representations of emotional states… an expression of ToM function”, the TAS-20 taps “a person’s difficulties in identifying and describing emotions to the extent that they are *aware* of their own deficits” (p. 402: emphasis added by the present authors), such that individuals with profound alexithymia and a lack of awareness of their emotional deficits would indeed fly under the TAS-20 radar. Though Lane et al. differentiate between the ability to describe an emotion and the ability to mentally represent an emotion, the former is associated with the TAS-20 and the latter with the LEAS (and with theory of mind); this view is not accepted by other theorists; in their review, Kashdan and colleagues [95], for instance, describe the LEAS as a test which measures “emotional awareness, the complexity of propositional knowledge of emotion”, and the TAS-20 as a test which captures “alexithymia, an impoverished *conceptual* system for emotion and emotion vocabulary” (p. 12: emphasis added by present authors). Maroti and colleagues conceptualize emotional awareness as a facet of alexithymia [111]; in a recent review of the construct, Goerlich [68], who does not even mention the LEAS, likewise describes alexithymia as a multifaceted construct. Present understanding is that these measures capture distinct but overlapping facets of “alexithymia” [111] and that their negligible overlap may reflect different neural mechanisms for the alexithymia facets, including emotional awareness; may reflect that behavioural and self-report measures frequently diverge; may reflect that emotion differentiation is quite separate from emotion perception; and/or may reflect poor self-awareness in the TAS-20. Our data does not offer conclusive suggestions to this long-standing debate, but it seems to suggest, so far as the relationship between autistic traits and eating psychopathology is concerned, that difficulty identifying emotions plays a particular role distinct from contributions of depression and anxiety.

### Limitations and future directions

We strove for statistical rigor and replicability with the testing of hypotheses across two experiments with separate participant groups. The standard alpha level (*p* < .05) has recently come under scrutiny [159–162], so we were reassured to see that central findings held when corrected to a more conservative .017 or even .007. Significant effect sizes supported these low *p* values, affording further confidence in our results. Consequently, we believe the data make important contributions to the literature around ED in autism and the role of alexithymia, including highlighting important avenues for future research—but there are several important caveats to consider and a great need for these findings to be reproduced in more controlled experimental settings.

Firstly, our cross-sectional design, though convenient for testing these initial hypotheses, disallows more rigorous scrutiny of causality and direction of these relationships. We cannot dismiss the alternative interpretation, for instance, that degree of alexithymia in our samples inflated the appearance of autistic traits (as might be supported by Hobson and colleagues [92]) and that autistic traits themselves predispose for eating difficulties. Neither can we dismiss the possible causal primacy of eating disordered behaviours giving rise to alexithymia (which, from then, might inflate autistic traits). With a lack of longitudinal research and that involving recovered individuals (alongside great variability around the definition of “recovered” [163]), the state dependency of alexithymia in ED is still highly uncertain. There is a suggestion that alexithymia in ED patients is independent of malnutrition and thus impervious to improvements in ED symptomatology (and alleviated depression) [81], which informs our tentative interpretation of causal primacy for alexithymia over ED symptomatology, but more rigorously controlled, longitudinal research is crucial to address these questions with greater certainty. The same is true for depression and anxiety, potential “mediator-outcome confounders” [134]. Whilst we could to an extent disentangle their contribution to the AQ–EAT-26 relationship from that of alexithymia, we could not speak to the causal primacy of alexithymia over depression and anxiety. Autistic traits are also highly associated with anxiety and depression [164], and it has been commonly assumed that as stable traits, autistic traits confer vulnerability to anxiety and depression. However, a directional influence of depression and anxiety on autistic traits could not, likewise, be dismissed.

Our sample is also limited in generalizability, being composed of British undergraduates (who would thus all have reasonable educational attainment levels); we did not collect ethnicity data, so the sample must be assumed to be largely Caucasian. The average age was low and the distribution of ages small. As aforementioned, the small male sample limits the confidence with which we can accept the null relationships seen in this group, though bootstrapping alleviates this issue to an extent. Furthermore, whilst investigation at trait or subclinical level within the general population is useful for hypothesis testing, the findings of the present study cannot be presumed generalizable to clinical populations: in this case, individuals with diagnosed autism and eating disorders. In populations with ED, previous research has established that alexithymia (high TAS-20 scores) increases the likelihood of an autism diagnosis [92]. Therefore, it would be necessary to examine whether heightened autistic traits, as commonly found with the AQ [43], are maintained when alexithymia is controlled for. Autistic traits are now commonly used as a proxy for autism spectrum conditions, but some question the suitability of this approach [72]. Whether alexithymia genuinely heightens risk of eating psychopathology longitudinally in females or males with autism; whether in the population of autistic people with ED, people with autism, and alexithymia are over-represented; and whether heightened levels of alexithymia are associated with greater severity of eating psychopathology in this group are important research questions.

Whilst online data collection affords many benefits, chiefly the possibility of accruing large datasets at minimal cost, future research might benefit from greater experimental control and operationalization of these variables beyond self-report questionnaires. Limitations of our measures must be recognized beyond those previously discussed around the TAS-20. In attempts to maintain our focus on alexithymia in its different facets, we chose not to deconstruct the AQ, though its factors seem differentially associated with the factors of the EAT-26 [34]. Concerning eating psychopathology, the measure employed in correlation analysis with autistic traits may also modulate the likelihood that significant relationships will be found. The EAT-26 is most popularly used in studies which purport a link between autistic traits and anorexia or ED [34], whereas the few studies using the EDE-Q, albeit in anorexic populations, did not find a relationship ([39, 45]; though see 144 for relationships between the AQ-short and the subscales of the EDE-Q). However, concerns have been raised that the EAT-26 may be prone to false positives in non-clinical samples such as ours: it was originally developed on a clinical sample, at a time prior to the inclusion of ED in the DSM, and is based on an outdated conceptualization that conflated AN and BN [165]. High scores in the EAT-26 could reflect any of a diverse group of “subclinical anorexics, partial syndrome anorexics, suspected anorexics, suspected bulimics, purgers, weight preoccupied and normal dieters” (Mintz and O’Halloran [163], p. 491). As such, although interest in the autism-ED relationship originated in behavioural similarities between autism and AN, with the EAT-26, we cannot claim to examine anything more specific than general eating psychopathology. Previous research has aimed for greater clarity by examining relationships between autistic traits and subscales of the EAT-26 [36], but this approach is contraindicated by one recent psychometric and factor analysis [104], which corroborates previous findings of unstable factorial structure, “weaving” of items between subscales, and sensitivity to variables as diverse as social pressure and awareness of nutritional content as conceptualized in orthorexia nervosa.

This emphasizes the need, if we wish to get closer to the specific type of eating psychopathology related to alexithymia and to autistic traits, to examine formally assessed clinical samples, diagnosed with rigorous adherence to DSM-5 [12] criteria for ED. It should perhaps be recognized, though, that many within the ED field take the view that “diagnoses are snapshots in the course of an eating disorder” (Fairburn and Cooper [166], p.9): that in the course of a lifetime, many patients move between presentations and may at times show mixed symptoms that defy categorization, especially if other psychiatric comorbidities are involved [167, 168]. The longitudinal course of ED in autistic individuals and those with high autistic traits is unknown but a crucial target for investigation. So too are additional variables that might be especially relevant to the autistic community, such as gender, given the prevalence of gender dysphoria in autistic people [169] and the heightened risk this seems to confer for eating psychopathology [170]. If overlaps might be seen between the two populations, pathogenic factors relevant for people with gender dysphoria [171] may be relevant for autistic people, too.

As a final note, it is critical to investigate the implications of experiencing alexithymia alongside an eating disorder for autistic individuals. Whilst alexithymia is associated with poorer prognosis in ED [81, 87] and has been linked by sufferers to their use of eating disordered behaviours [146], the question is open as to the effects of comorbid alexithymia on prognosis for autistic individuals with ED. In a similar vein, though alexithymia is associated with depression and suicidality in people with ED [172–174] as well as in psychiatric populations in general [82, 83], the contribution of alexithymia to the heightened suicide rates seen in autism [175] is unknown, as are the effects of autistic traits or autism with or without alexithymia on the suicide rates in ED. The next step, of course, is to ascertain what therapeutic modifications would be most appropriate to individuals experiencing both autism and alexithymia alongside an ED. Recent work has suggested that, given the understanding that alexithymia encapsulates emotional and more physiological interoceptive difficulties, interventions which increase awareness of physiological arousal associated with emotional experiences may be helpful [176], as might therapeutic work supporting cognitive reappraisal [86].

## Conclusions

The present research highlights a yet unconsidered factor in the well-reported link between autism and eating psychopathology: alexithymia, a variable which may be of considerable import in the heightened risk, treatment, and prognosis of individuals experiencing an ED alongside autism, but which requires further theoretical clarification for fuller comprehension and utility. Our findings showed that alexithymia, most particularly difficulties identifying emotions, may partially explain the prevalence of eating disorder symptomatology in females with high autistic traits. To see whether this finding also explains the prevalence of ED in autistic people requires replication and controlled experimental scrutiny in clinical as well as non-clinical samples. Our findings also emphasize the need to consider sex as a variable which may modulate the risk factors that lead to ED in autistic and non-autistic men and women.

## Supplementary information

**Additional file 1:.** Supplementary item 1: alpha coefficients for measures from Experiments 1 and 2. Supplementary item 2: LEAS scoring. Supplementary item 3: Descriptive statistics and mediation statistical notations from the additional analysis controlling for anxiety and depression (Experiment 2).

## Data Availability

The datasets used and/or analysed during the current study are available from the corresponding author on reasonable request.

## Abbreviations

ADOS-2: Autism Diagnostic Interview Revised (version 2)
AN: Anorexia nervosa
AQ: Autism-Spectrum Quotient
ARFID: Avoidance/restrictive food intake disorder
ASC: Autism spectrum conditions
BMI: Body mass index
BN: Bulimia nervosa
DIF: Difficulty Identifying Feelings, a subscale of the TAS-20
DDF: Difficulty Describing Feelings, a subscale of the TAS-20
DSM: Diagnostic and Statistical Manual for Mental Disorders
EAT-26: Eating Attitudes Test (26 item version)
ED: Eating disorders
EOT: Externally Oriented Thinking, a subscale of the TAS-20
LEAS: Levels of Emotional Awareness Scale
TAS-20: Toronto Alexithymia Scale (20 item version)

## Footnotes

1 With some theorists preferring to talk about ‘the autisms’, it is likely that ASC encapsulates a family of genetically heterogenous conditions with these common presenting features [13]

2 It is relevant to note that the prevalence of eating disorders is also heightened in people with other neurodevelopmental conditions, namely ADHD [50]. As autism and ADHD are strongly comorbid and share genetic heritability [51], individuals with both autism and ADHD might possibly be found at higher rates in the eating disorder population than individuals with either ADHD or autism alone—this is yet to be explored.

3 Some studies do, however, find differences between ED patient groups and control comparison groups on the LEAS [119, 120], so there is some discrepancy in the field.
