# Supplementary material for: Alexithymia may explain the relationship between autistic traits and eating disorder psychopathology
Source: Mol Autism. 2020 Aug 5;11:63. doi: 10.1186/s13229-020-00364-z (PMC7406391; doi:10.1186/s13229-020-00364-z)
Supplement: Supplementary file 1 — Additional file 1:. Supplementary item 1: alpha coefficients for measures from Experiments 1 and 2. Supplementary item 2: LEAS scoring. Supplementary item 3: Descriptive statistics and mediation statistical notations from the additional analysis controlling for anxiety and depression (Experiment 2). [file 13229_2020_364_MOESM1_ESM.docx]

**Supplementary materials**

*Table of contents:*

1. Alpha levels for TAS-20, AQ and EAT-26 (Experiments 1 and 2)
2. LEAS scoring (Experiment 2)
3. Descriptive statistics and mediation statistical notations from the additional analysis controlling for anxiety and depression (Experiment 2)

**Supplementary item 1: alpha coefficients for measures from Experiments 1 and 2**

*Experiment 1*

| **Test** | **Alpha levels** |
| --- | --- |
| Autism Spectrum Quotient (AQ): Total | α = .869 |
| Toronto Alexithymia Scale (TAS-20): Total | α = .852 |
| Eating Attitudes Test (EAT-26): Total | α = .885 |

*Experiment 2*

| **Test** | **Alpha levels** |
| --- | --- |
| Autism Spectrum Quotient (AQ): Total | α = .829 |
| Toronto Alexithymia Scale (TAS-20): Total  *DIF*  *DDF*  *EOT* | α = .860  α = .871  α = .818  α = .526 |
| Eating Attitudes Test (EAT-26): Total | α = .913 |

**Supplementary item 2: LEAS scoring**

In our use of the LEAS-B, we followed the scoring manual of the second edition [197], which, as explained in the manual, has been shown to possess good psychometric properties across a number of studies. In each of the 10 scenarios presented, when asked how they would feel and how another person would feel, individuals consequently receive a score of zero if they describe cognitive processes or states rather than emotions (e.g. “I would expect *____* to happen”, “I would feel confused”), a score of 1 if they describe a bodily or physical sensation (e.g. “I would feel hungry”), a score of 2 if they describe an action with no clear emotional state (e.g. “I would tell him to get out”) or an emotion state that is vague or non-specific (e.g. “I would feel bad”), a score of 3 for any a single but precise emotion (e.g. “I would feel angry”), and a score of 4 for describing two distinct emotions that are not synonymous (e.g.: “I would feel anxious but excited”). Notably, the emotions described do not need to conform to what most people might feel in these scenarios, and nor the perceived complexity of the emotional reaction be scored: only the explicit statements. Each scenario requires an answer both for the self and for the other person in the scenario, such that the test generates a Self Score and an Other Score for each item, which are taken to form a total Score for that item, and these item scores are summed to provide a single score across the 20 items on the LEAS. To insure inter-rater reliability, two researchers (ZC and ART) scored the items individually and then compared their scores, reaching a high overall agreement (89%), which is in agreement with what is reported in the literature [175].

The lower the score in the LEAS, the poorer emotional awareness. Scores in the 0-10 range would indicate LEAS Level 1 (awareness of bodily sensation); scores between 10-20 translate to LEAS Level 2 (action tendency and undifferentiated affect state); scores between 20-30 indicate LEAS Level 3 (single emotion); scores between 30-40 reflect LEAS Level 4 (blend of emotions); and scores 40-50 translate to LEAS Level 5 (blend of blends of emotions). There is no defined cut-off point for alexithymia, but non-alexithymic individuals in the general population score an average total of 61.9 on the 20 item version [106]. The short version (10 items) is statistically parallel to this, so the average score is 30.95 in the general population.

**Supplementary item 3: Descriptive statistics and mediation statistical notations from the additional analysis controlling for anxiety and depression (Experiment 2)**

The descriptive statistics were re-calculated for this subset of participants. These averages are as follows:

| **Subset with depression and anxiety as covariates** | All participants (n = 233) | Participants (f/m) scoring at/above cut-offs, or below LEAS average: |
| --- | --- | --- |
| Autistic traits (AQ) | 16.6 (7.1), *45* | 20 f / 4 m |
| EAT-26 Total | 12.4 (13.4), *75* | 42 f / 3 m |
| TAS-20 Total | 50.5 (12.4), *62* | 16 f / 0 m |
| TAS-20 DDF | 14.4 (5), 20 | - |
| TAS-20 DIF | 16.9 (6.9), 26 | - |
| TAS-20 EOT | 19.2 (3.9), 24 | - |
| LEAS | 31.8 (3.9), *23* | 76 f / 21 m |
| PHQ-9 | 9.26 (6.7), *27* | 16 f / 0 m |
| BAI | 20.9 (13.8), *59* | 16 f / 0 m |

Average scores, followed by standard deviation (brackets) and range (italics) for male and female participants on the Autism-Spectrum Quotient (AQ), Eating Attitudes Test-26 (EAT-26)), Toronto Alexithymia Scale-20 (TAS-20 total and subscales), Levels of Emotional Awareness Scale (LEAS), Patient Health Questionnaire (PHQ-9), and Beck Anxiety Inventory (BAI). Participants (female / male) who scored above clinical cut-offs in both analyses are indicated in the fourth column. Cut-offs are 26 for autistic traits; 20 for eating psychopathology; 61 for alexithymia as measured by the TAS-20; 3.2 for depression in this age range; and 7.3 for anxiety in this age range; and below the general population average for the LEAS (31).

*DDF, DIF and EOT as parallel mediators, depression (PHQ) and anxiety (BAI) as covariates (Model 4):*

Autistic traits were significantly related to DDF (b = .22, p < .001; *R^2^* = .28, *F* (3, 219) = 28.06, *p* < .001), with significant effects of depression (b = .18, p = .0023) but not anxiety (p = .24) on this relationship. Autistic traits were significantly related to DIF (b = .23, p = .001; *R^2^* = .40, *F* (3, 219) = 49.48, *p* < .001), with significant effects of depression (b = .26, p = .0007) and anxiety (b = .15, p = .0001) on the relationship. Furthermore, autistic traits were significantly related to EOT (b = .15, p = .003; *R^2^* = .06, *F* (3, 219) = 4.68, *p* = .0035), but there were no effects of depression (p = .1374) or anxiety (p = .8721) on this relationship.


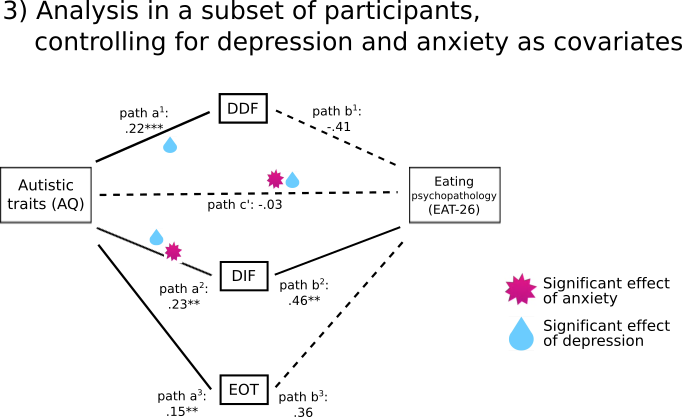


Regression coefficients for relationship between autistic traits (AQ), TAS-20 subscales and eating psychopathology (EAT-26). P values are reflected by asterisks, where p < .001 is depicted as ***, and p < .01 is depicted with **.

The direct effect of autistic traits on EAT-26 scores was non-significant (b = -.03, p = .8207; *R^2^* = .33, *F* (6, 216) = 17.49, *p* < .001); depression (b = .71, p < .001), anxiety (b = .15, p = .0468) and DIF (b = .46, p = .0079) were all significant contributors to the model. Relationships between DDF, EOT and EAT-26 scores were marginally non-significant (DDF: b = -.41, p = .0639; EOT: b = .36, p = .0869). Of the three subscales, only the indirect effect of DIF was significant (b = .11, CI: .0280, .2064). Confidence intervals for DDF (b = -.09, CI: -.1890, -.0069) and EOT (b = .05, CI: -.0068, .1185) suggested that these subscales were not significant mediators of the relationship between autistic traits and eating psychopathology.

*LEAS as mediator, depression (PHQ) and anxiety (BAI) as covariates (Model 4):*

The relationship between autistic traits and LEAS scores remained non-significant (p = .1556). Neither were anxiety (p = .1477) or depression (p = .1818) related to LEAS scores.

The direct effect of autistic traits on EAT-26 scores was non-significant (b = .04, p = .7507; *R^2^* = .30, *F* (4, 218) = 22.97, *p* < .001), the variance in the model was accounted for by depression (b = .72, p < .001) and anxiety (b = .21, p = .0059), without a significant indirect effect of LEAS scores (b = .0026, CI: -.0214, .0501).
